# Prospective randomized study on the effect of music on anxiety and pain related to CT-guided percutaneous interventions

**DOI:** 10.1007/s00330-025-11441-3

**Published:** 2025-02-25

**Authors:** Florian Nima Fleckenstein, Kara Ann Hecker, Florian Schusta, Anna Pöhlmann, Timo Alexander Auer, Bernhard Gebauer, Federico Collettini

**Affiliations:** 1 Charité—Universitätsmedizin Berlin, Corporate Member of Freie Universität Berlin and Humboldt-Universität zu Berlin, Department of Diagnostic and Interventional Radiology, Berlin, Germany; 2 Berlin Institute of Health at Charité—Universitätsmedizin Berlin, BIH Biomedical Innovation Academy, BIH Charité Clinician Scientist Program, Berlin, Germany; 3 Charité—Universitätsmedizin Berlin, corporate member of Freie Universität Berlin and Humboldt-Universität zu Berlin, Institute of Biometry and Clinical Epidemiology, Berlin, Germany

**Keywords:** Interventional radiology, Biopsy, Drainage, Pain management, Music therapy

## Abstract

**Background:**

CT-guided percutaneous interventions may cause patients to experience high levels of stress and anxiety, negatively affecting post-interventional outcomes. Music played during medical interventions has been shown to reduce anxiety and pain, yet the effect of music on patients undergoing interventional radiology procedures has not been evaluated. The aim of this study was to assess whether music played during CT-guided percutaneous interventions may reduce anxiety and pain.

**Methods:**

This prospective randomized controlled trial included patients undergoing CT-guided transcutaneous procedures. The final analysis included a total of 209 patients, randomized into a music group (MG, *n* = 107) and a control group (CG, *n* = 102). Items of state and trait anxiety were analysed using the short form of the State Trait Anxiety Inventory (STAI-6) before and after the procedure. Post-procedural pain was assessed with the numeric rating scale (NRS) with faces.

**Results:**

Patients in the MG exhibited lower anxiety and a greater reduction in anxiety levels as compared to patients in the CG (*p* < 0.001, respectively). The median change of anxiety was 6.5 ± 3.8 (range: −3 to 14) in the MG versus 3.7 ± 3.5 (range: −6 to 13) in the CG. Post-procedural pain showed a value of 2 ± 2.1 (median, range: 0 to 9) in the MG, versus 6 ± 2.4, (median, range: 0 to 10) in the CG.

**Conclusion:**

Exposure to music during CT-guided percutaneous interventions can aid in significantly lowering peri-interventional anxiety and pain and thus improve overall patient care without any negative side effects.

**Key Points:**

***Question*** *Does listening to music during CT-guided percutaneous interventions reduce peri-interventional anxiety and pain in patients?*

***Findings*** *This prospective randomized-controlled trial found that patients exposed to music during CT-guided interventions experienced significantly lower levels of anxiety and pain compared to those in a control group.*

***Clinical relevance*** *Incorporating music into CT-guided interventions provides an easy, non-invasive, and cost-effective method to reduce patient anxiety and pain in the clinical setting.*

## Introduction

Computed tomography (CT)-guided interventions form a mainstay in the field of interventional radiology (IR) enabling common diagnostic and therapeutic procedures such as tumor biopsy, abscess drainage, and tumor ablation [1]. The minimal-invasive character of these interventions holds numerous advantages, including a low risk of complications and an efficient procedure time. However, since most of these procedures are performed on patients with full consciousness, pain, and anxiety management pose a particular challenge in the field of IR.

Patients who find themselves in a sterile and foreign surroundings awaiting to undergo invasive medical interventions show high levels of pre-procedural stress and anxiety [2]. Anxiety may, in turn, alter the perception of pain by lowering the pain-threshold, which results in an increased use of analgesics and sedatives, lengthening recovery and hospital discharge time and thus, hampering post-therapeutic outcomes [3, 4]. Moreover, a decrease in cellular immunity due to cortisol-triggered pro-inflammatory processes may prolong recovery and healing [3, 5]. The goal of optimal patient care is to diminish peri-procedural anxiety and pain and to reduce suffering as much as possible. Inadequate treatment or negligence of pain can lead to higher morbidity and mortality as well as chronic pain. This can extend hospitalization and recovery time and cause complications [5, 6]. Analgesic medications such as opioids and NSAIDs can also have severe side effects when over-dosed or misused, thus leading to follow-up diseases [7–9]. Therefore, interventions to reduce stress, anxiety, and pain associated with IR may contribute to improved post-interventional outcomes and overall patient care.

Music has been shown to activate multiple reward centers in the central nervous system and have a direct impact on hormone levels by lowering blood cortisol and thereby promoting relaxation [10]. When listening to music, a shift from sympathetic to parasympathetic activity is detected, leading to high levels of parasympathetic activity and a decrease in nerve activity in sympathetic neurons [11, 12]. More specifically, in the context of peri-procedural outcomes, three separate effects of music have been observed: emotional comfort, distraction from pain, and the ability to counteract feelings of subjection and loss of control by acting as a familiar stimulus in the foreign hospital environment [4, 13]. These effects have instigated an interest in musical interventions in the medical context, with several studies demonstrating a positive effect of music on pre-interventional anxiety and the threshold and perception of peri-procedural and chronic pain [14–16]. The altered pain perception possibly derives from modified endogenous opioid levels [10, 11]. Although the mechanisms by which music affects these processes are yet unclear [11], these findings point toward a beneficial effect of music interventions on several issues related to pain and anxiety.

In this context, music could serve as an additional pillar in perioperative analgesia to reduce stress and anxiety levels and even reduce the amount of analgetic medication. In addition, music constitutes an inexpensive, non-invasive, easily available instrument that does not interact with medication and is uncomplicated in its use [11, 17, 18]. Despite the existence of multiple studies that tested the effect of music on pre- and post-interventional anxiety and pain [19], the effect of music on patients undergoing CT-guided percutaneous interventions has not yet been evaluated.

In this prospective randomized study, we investigate the effects of music on the perception of pain and anxiety during and after CT-guided percutaneous interventions.

## Materials and methods

Consecutive patients referred for CT-guided interventions were considered for inclusion in the study. The study was approved by the local ethics committee (EA4/079/16) and registered with the German Clinical Trials Register (no. DRKS00026003 accessible under https://www.drks.de/). Inclusion criteria were indication for diagnostic or therapeutic CT-guided procedures (e.g., tumor biopsy, abscess drainage, or tumor ablation), signed informed consent, and ability to read and understand German language. Patients with mental impairment, significant hearing difficulties, or those who underwent the procedure under general anesthesia were not included in the study. Eligible participants were randomly assigned (1:1) to either a music or control group (CG) using a computer-generated random number of series (https://www.randomizer.org/). Before the start of the procedure, participants were asked to indicate their favorite music to be played during the procedure. Participants could choose among selected pop, jazz, classical, or rock playlists. If the participant did not specify his/her taste in music, the physician was allowed to choose the style of music. During the procedure, music was played through a speaker at a volume ranging from 50 to 60 dB [13] for subjects in the music group (MG), whereas patients in the CG underwent the procedure in the same setting without music. Prior to the procedure, a detailed explanation of the intervention was provided. For each participant, a German version of the state anxiety questionnaire (State-Trait Anxiety Inventory [STAI]) and a Wong-Baker FACES Pain Rating Scale were administered before and after the procedure.

### State-Trait Anxiety Inventory (STAI)

The STAI is a psychometric questionnaire developed by Spielberger et al in 1981 to differentiate between self-assessed state and trait anxiety [20, 21]. In the present study, a shortened version of the original 20-item STAI, the STAI-6, was used, containing “the three highest anxiety-present and anxiety-absent items” [22]. The STAI-6 was chosen to ensure both high validity and reliability, while also shortening the amount of time needed pre- and post-intervention to complete the inventory [22, 23]. A German version of the STAI-6 was used based on the current “Form Y” of the German 20-item STAI established in 1983 [24]. Frequencies of post-procedural items connotated with negative (tense, upset, concern) and positive (calm, relax, content) emotions were compared between the two groups using the Mann–Whitney *U*-test. Each statement could be ranked as “not at all”, “somewhat”, “considerably”, or “very much”. To evaluate the reduction of anxiety levels, the mean ranks, and the median “anxiety change scores” in both groups were compared.

### Wong-Baker FACES Pain Rating Scale

The second psychometric tool employed in this study was the Wong-Baker FACES Pain Rating Scale, a numeric rating scale (NRS) combined with faces. This self-reported scale ranges pain between 0 and 10 to evaluate the subjectively experienced level of pain after the procedure. By choosing a smiley ranging from “happy face” (0) to “crying face” (10) and a scale from “no pain” (0) to “worst pain” (10), as well as written descriptions, patients can easily communicate their level of pain.

### Statistical analysis

SPSS Statistics (Version 27.0 for Windows, IBM) was used to conduct the statistical analysis. Descriptive statistics were employed to determine absolute and relative frequencies, medians, and ranges. The normality of metric variables was evaluated through graphical methods. The Mann–Whitney *U*-test was used to compare ordinal variables between both groups in addition to estimated relative effects.

An “anxiety change score” was calculated for both the IC and CG groups to evaluate differences in anxiety levels before and after the intervention. To derive this score, the three negatively connoted items (out of six total items) were reversed, and all items were summed for each individual. Positively connoted items were assigned values ranging from 1 to 4, while negatively connoted items were assigned values ranging from −1 to −4. This process resulted in pre- and post-intervention anxiety scores ranging from −9 to 9 per person. The anxiety change score was then calculated by subtracting the post-intervention score from the pre-intervention score, yielding possible values between −18 and 18. Negative scores indicate an increase in anxiety, while positive scores reflect a reduction. Differences in anxiety change scores and pain levels between the MG and CG groups were analyzed using the Mann−Whitney *U*-test, and the interquartile range (IQR) was calculated for better comparison. All *p*-value are interpreted in an exploratory manner, and with that, no adjustment for multiple testing is made.

## Results

**Fig. 1:**
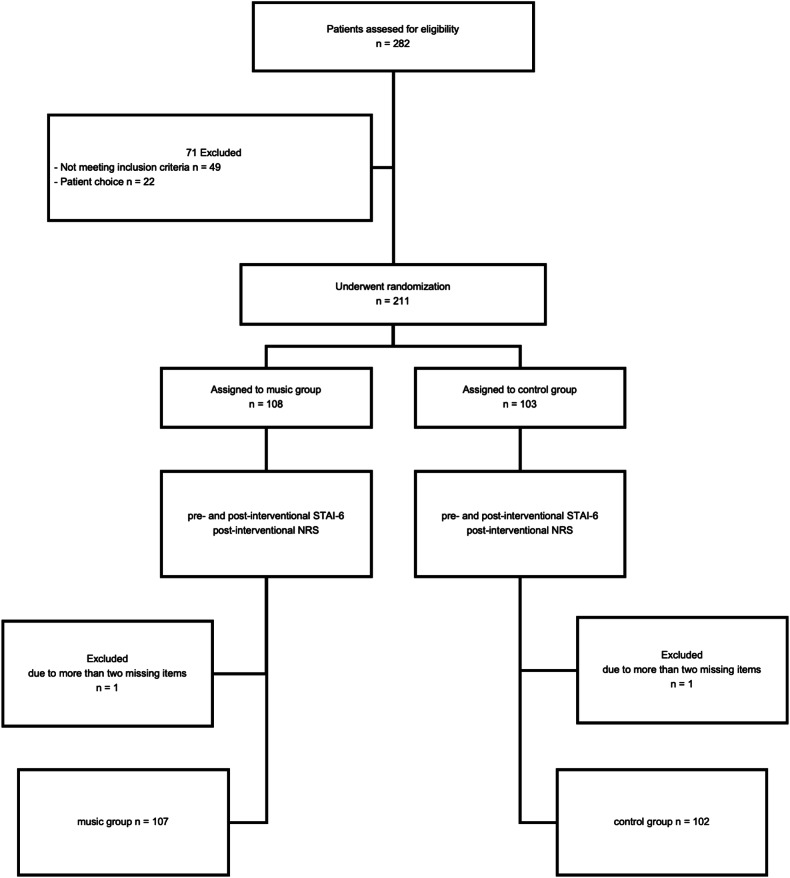
Study flow-chart. STAI, State-Trait Anxiety Inventory; NRS, numeric rating scale

Participants were recruited between January 1st and August 31st, 2021. 282 patients were assessed for eligibility. 211 patients meeting the inclusion and exclusion criteria were included in the study and randomized in each group (MG, *n* = 107; CG, *n* = 102). Two participants (one in each group) were excluded from the final analysis because more than two items in their inventories were missing. Hence, the final analysis comprised data from 209 participants (Fig. 1; MG, *n* = 107; CG, *n* = 102).

Participants’ baseline characteristics and procedural details are described in Table 1. Participant’s baseline characteristics were similar in both groups. Overall, 127 male (MG: *n* = 64, CG: *n* = 63) and 82 female participants (MG: *n* = 43, CG: *n* = 39) with a mean age of 62.1 ± 13.9 years (MG: 63.7 ± 13.8 years, CG: 60.5 ± 14.0 years) were included.

**Table 1:** Baseline characteristics and procedural details

Parameter	MG (*n* = 107)	CG (*n* = 102)	Total (*n* = 209)
Sex, *n* (%)			
Male	64 (60)	63 (62)	127 (61)
Female	43 (40)	39 (38)	82 (39)
Age (years), mean ± SD	63.7 ± 13.8	60.5 ± 14.0	62.1 ± 13.9
Underlying condition			
Infectious disease, *n* (%)	16 (15)	15 (15)	31 (15)
Oncologic disease, *n* (%)	65 (60)	70 (68)	135 (65)
Pain disorder, *n* (%)	17 (16)	11 (11)	28 (13)
Other disease, *n* (%)	10 (9)	6 (6)	15 (7)
Procedures			
CT-guided Brachytherapy, *n* (%)	4 (4)	9 (9)	13 (6)
Drainage, *n* (%)	34 (32)	34 (33)	68 (33)
Extradural injection, *n* (%)	1 (1)	1 (1)	2 (1)
Gastropexy/gastrostomy, *n* (%)	8 (7)	4 (4)	12 (6)
Gold marker insertion, *n* (%)	3 (3)	2 (2)	5 (2)
Liver puncture, *n* (%)	9 (8)	12 (12)	21 (10)
Lung puncture, *n* (%)	12 (11)	14 (14)	26 (12)
Other puncture, *n* (%)	20 (19)	14 (14)	34 (16)
Periradicular therapy (PRT), *n* (%)	16 (15)	11 (10)	27 (13)
Radiofrequency ablation (RFA), *n* (%)	0 (0)	1 (1.0)	1 (1)
Familiarity with the intervention, *n* (%)			
Yes	16 (15)	14 (14)	30 (14)
No	91 (85)	88 (86)	179 (86)
Procedural time (in minutes), mean ± SD	10.6 ± 7.5	10.9 ± 8.1	10.8 ± 7.8
Complications, *n* (%)			
Yes	2 (2)	4 (4)	6 (3)
No	105 (98)	98 (96)	203 (97)

In both study arms, the majority of participants underwent CT-guided interventions due to oncologic disease (MG: 65/107, and CG: 70/102). Music genres chosen in the MG are shown in Supplementary Fig.  1.

### State-Trait Anxiety Inventory (STAI)

**Fig. 2:**
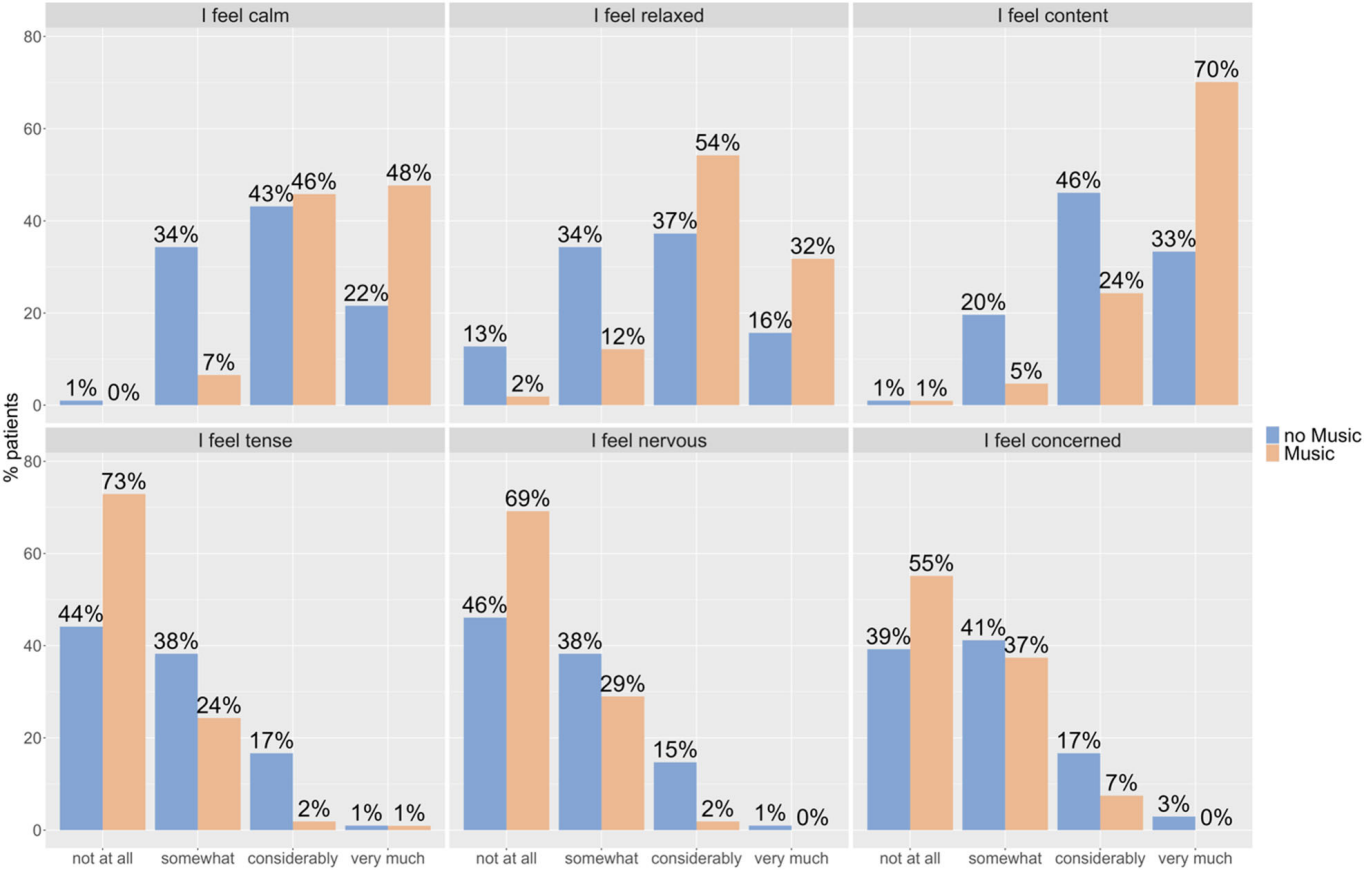
Bar diagram showing frequencies of post-interventional items connotated with positive and negative emotions

Every pre-interventional item in both the MG and CG initially showed the same median value, thus creating an equal basis for comparison. Pre-interventional STAI values are shown in Supplementary Fig.  2. The distribution of the frequencies of all post-interventional items differed considerably between the MG and CG groups, indicating lower post-operative anxiety levels in the MG (see Fig. 2, A. calm: *ψ* = 0.70, *p* < 0.001; B. relaxed: *ψ* = 0.68, *p* < 0.001; C. content: *ψ* = 0.69, *p* < 0.001; D. tense:* ψ* = 0.34, *p* < 0.001; E. nervous: *ψ* = 0.36 *p* < 0.001; F. concerned: *ψ* = 0.40, *p* < 0.05, Mann–Whitney-*U*-test). Also, the relative effects *ψ* show that the MG tends to have higher values in the first three (positively connoted) items than the MG, and lower values in the three negatively connoted items.

**Fig. 3:**
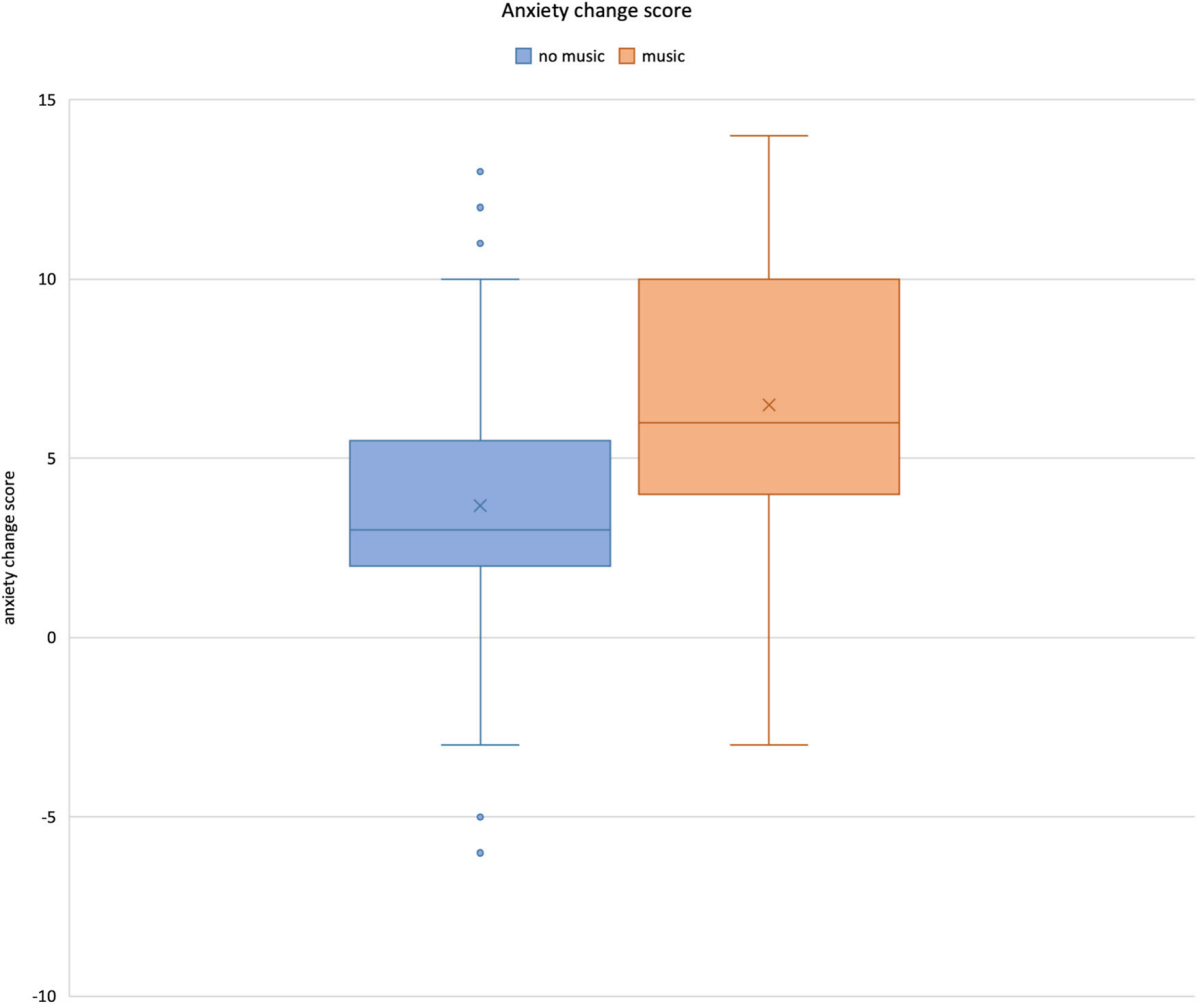
Boxplots of anxiety change score values for group comparison. X marks the mean value

The median “anxiety change score” in the MG was 6.5 ± 3.8 (range: −3 to 14, IQR 6), in the CG it was 3.7 ± 3.5 (range: −6 to 13, IQR 3.5). The reduction of state anxiety levels was greater in the MG due to the bigger change of values towards ranks that relate to lower anxiety (Fig. 3, *ψ* = 0.32, *p* < 0.001, Mann−Whitney *U*-test). Anxiety levels were also reduced in the CG, yet to a considerably smaller extent.

### Wong-Baker FACES Pain Rating Scale

**Fig. 4:**
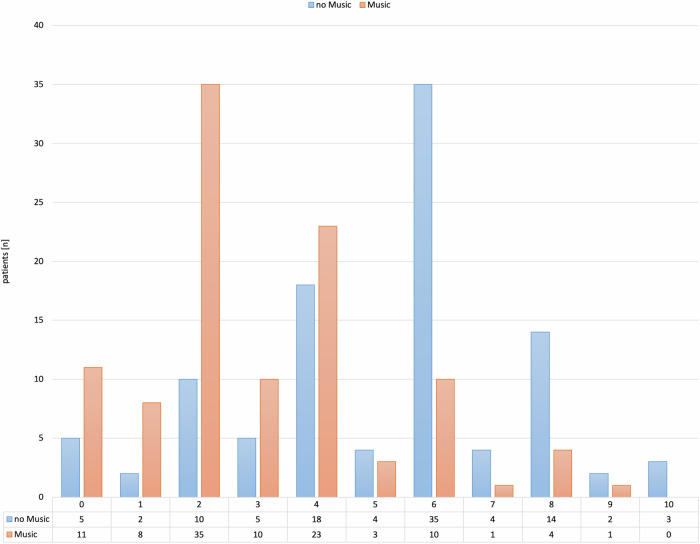
Pain level as NRS value distribution in both groups shown as bar diagrams. NRS, numeric rating scale

Patients in the MG rated their pain with a median value of 2 (mean: 3.1 ± 2.1; range: 0 to 9) on the NRS with Faces scale, while patients in the CG ranked their pain with a median value of 6 (mean: 5.2 ± 2.4, range: 0 to 10; Fig. 4). For 81.3% of patients in the MG, the pain level ranged from 0 to 4, while only 39.2% of patients in the CG rated their pain in this range. The pain levels differed relevantly between both groups (*ψ* = 0.25, *p* < 0.001, Mann–Whitney *U*-test).

## Discussion

The results of this study show that post-procedural anxiety levels were considerably lower in the MG as compared to the CG and, therefore, reduced more in the MG than in the CG. Moreover, the subjective level of post-interventional pain was lower in the MG as measured by NRS with Faces.

Consistent with findings from other studies [19, 25–30], our results demonstrate that music can effectively reduce anxiety and pain in the perioperative setting. The use of the STAI-6 scale provides a standardized and detailed method for evaluating how individual items influence anxiety levels in general. Yet, analyzing changes in specific item values allows for the identification of precise factors contributing to the overall reduction in anxiety levels.

Interestingly, in the MG, the IQR of anxiety reduction was larger than in the CG (Fig. 4). These interindividual differences might show that the effectiveness of the peri-interventional use of music might depend on baseline conditions that vary from patient to patient. To optimize the use of music as a stress-relieving element in the future, an assessment of patients’ general music listening habits, along with other underlying factors, could enable a pre-interventional, individualized evaluation of music’s efficacy during these procedures. To come into full effect, factors such as a pleasant room temperature, pleasant volume while reducing background noise, and a comfortable body position during the intervention, are of utmost importance [31].

In our study, the participants of the MG experienced considerably less pain than those in the CG. This leads to the conclusion that music may serve to improve the pain-threshold of patients and to reduce the peri- and post-interventional need for analgesics [11]. The pre- and peri-interventional use of music could help patients to decide in favor for an intervention under local anesthesia rather than undergoing general anesthesia, thanks to reduced anxiety and pain beforehand [3]. The smaller the amount of pain, the lower the required dose of analgesics needed to treat the patient and hence, the lower the number of side effects and interactions with other pharmaceuticals and the faster the recovery. This improves overall patient care by potentially shortening hospitalization time, lowering costs, and preventing complications as well as chronic pain, as demonstrated in publications focused on procedures outside of interventional radiology [5, 15].

A majority of patients in our study were battling cancer. It is especially crucial for these individuals to focus on improving their quality of life and alleviating psychological stress. Often, these patients endure prolonged periods of pain and undergo challenging treatments in settings they are not accustomed to. This frequently leads to feelings of losing control over their own bodies and decisions, a direct consequence of their illness. In light of this and drawing from our findings, the incorporation of music stands out as a beneficial element in supplementary therapy for pain and anxiety [3, 13].

The number of participants is high compared to former studies that tested the effects of a peri-procedural use of music, although several limitations of our study must be taken into consideration. Our statistical analysis of the secondary outcome, post-procedural pain, must be considered explorative because the *p*-value could not be adjusted for multiple testing. Despite this, the test showed a highly effective power. Regarding the course of the study, the Hawthorne effect could have biased the results and weakened internal validity, because blinding was not possible due to the nature of the music intervention. The study was conducted at a single hospital department, thus decreasing external validity and future studies at multiple intervention centers should be conducted to confirm the results. Finally, as an alternative to the STAI-6, the 20-item STAI could achieve a more comprehensive overall assessment of anxiety features since more anxiety-defining items are being evaluated.

Building on the findings of this study, future research could benefit from comparing the effectiveness of music with other non-pharmacological methods, such as hypnotherapy or virtual reality distraction techniques, while assessing the potential impact on medical staff [10, 32]. Addressing these aspects in future studies will provide a more comprehensive understanding of the broader benefits of music therapy and its role in creating a supportive procedural environment.

In conclusion, music serves as an easily implementable, cost-free, and non-intrusive method to lessen anxiety and pain during minimal-invasive, CT-guided interventions. Beyond mere statistical significance, we are of the opinion that these results hold substantial clinical importance, extending well past the realm of IR.

## Supplementary information

ELECTRONIC SUPPLEMENTARY MATERIAL

## Abbreviations

CG: Control group
IQR: Interquartile range
MG: Music group
NRS: Numeric rating scale
STAI-6: State Trait Anxiety Inventory
