# Supplementary material for: Prospective randomized study on the effect of music on anxiety and pain related to CT-guided percutaneous interventions
Source: Eur Radiol. 2025 Feb 25;35(9):5826–34. doi: 10.1007/s00330-025-11441-3 (PMC12350575; doi:10.1007/s00330-025-11441-3)

Prospective randomized study on the effect of music on anxiety and pain related to CT-guided percutaneous interventions

ELECTRONIC SUPPLEMENTARY MATERIAL

Supplementary Figure 1: *Choices of music genre played in the music group (MG, n=107)*

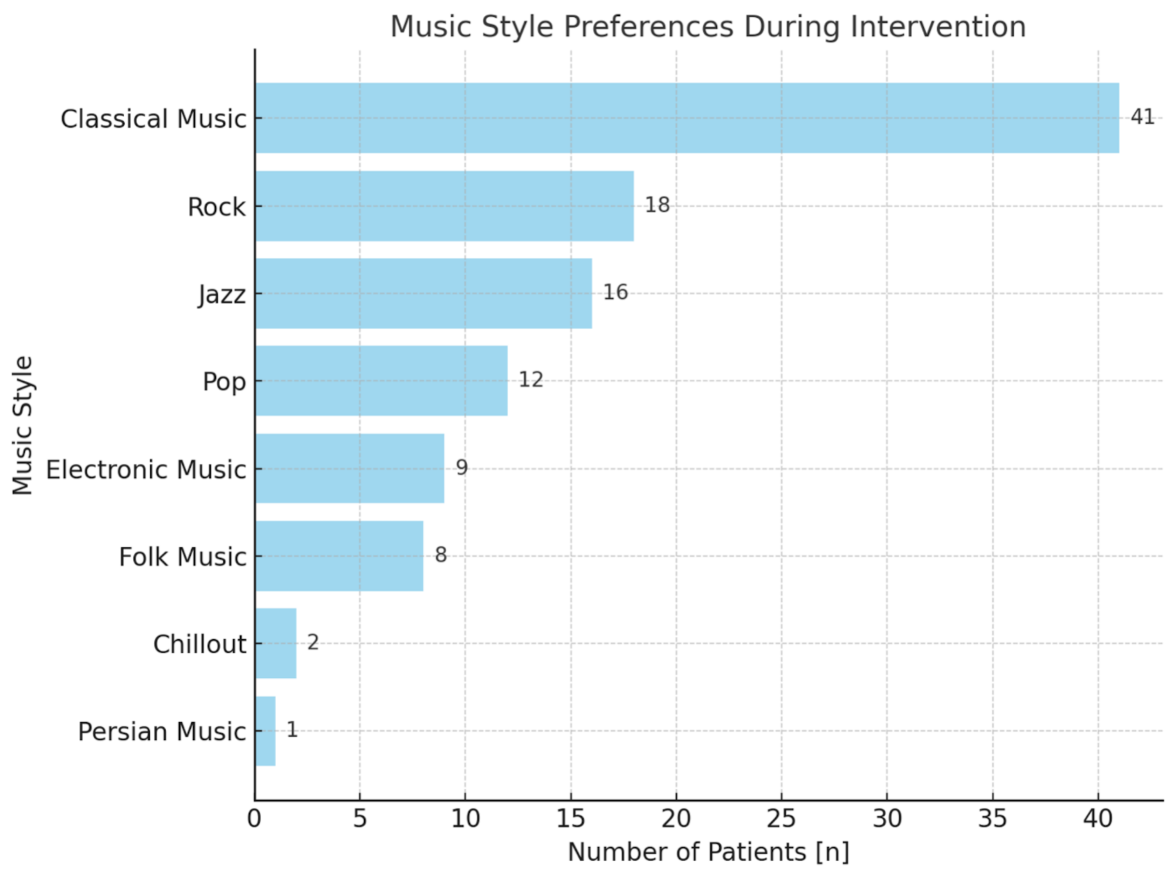

Supplementary Figure 2: *Frequencies of pre-interventional items connotated with positive and negative emotions.*

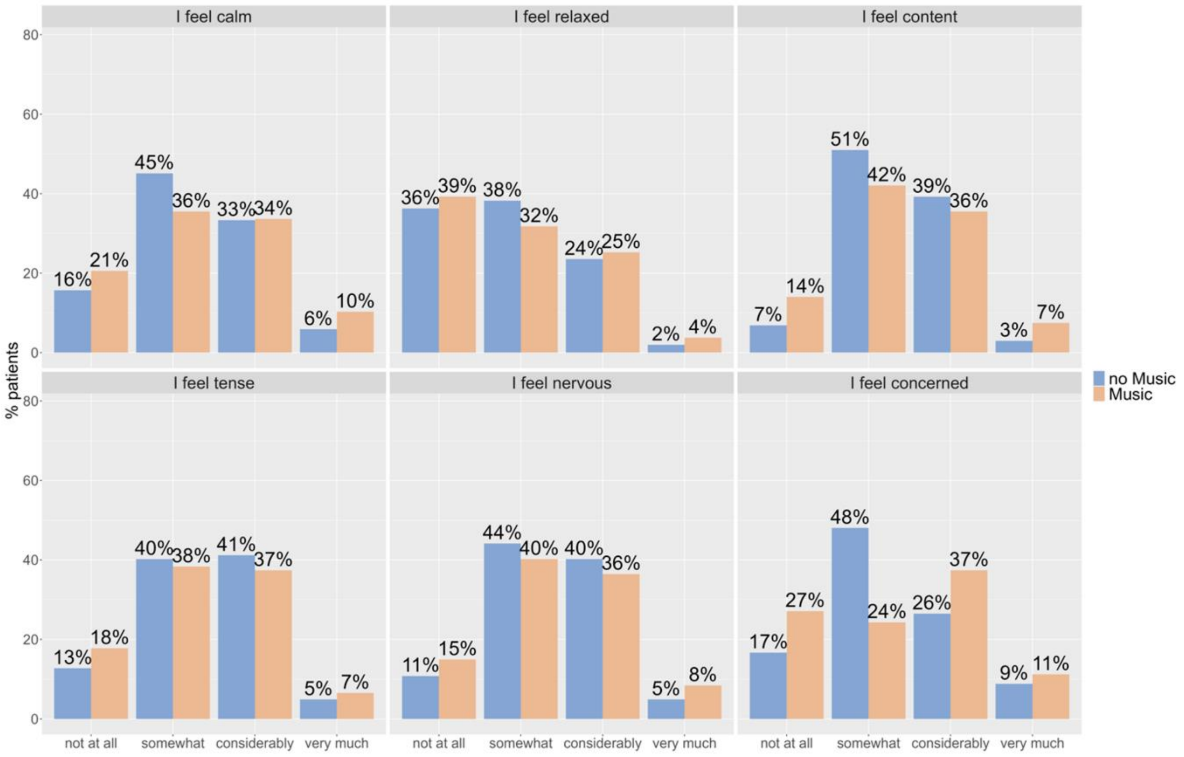

Supplement: Supplementary file 1 — ELECTRONIC SUPPLEMENTARY MATERIAL [file 330_2025_11441_MOESM1_ESM.pdf]
